# Draft Genome Sequences of Six Bordetella hinzii Isolates Acquired from Avian and Mammalian Hosts

**DOI:** 10.1128/genomeA.00081-15

**Published:** 2015-03-19

**Authors:** Karen B. Register, Yury V. Ivanov, Eric T. Harvill, Lauren Brinkac, Maria Kim, Liliana Losada

**Affiliations:** a National Animal Disease Center, USDA, Agricultural Research Service, Ames, Iowa, USA; b Department of Veterinary and Biomedical Sciences, The Pennsylvania State University, University Park, Pennsylvania, USA; c J. Craig Venter Institute, Rockville, Maryland, USA

## Abstract

Bordetella hinzii is a Gram-negative bacterium known to infect poultry, humans, rabbits, and rodents. It is an opportunistic pathogen in immunocompromised humans, and some strains cause mild to moderate respiratory disease in turkeys. Little is known as to the degree of genetic diversity within the species or the genetic basis for virulence. Here, we report the genome sequences of six isolates of B. hinzii acquired from humans, rabbits, or turkeys. These data provide a framework for refining the population structure of the genus, establishing relationships among genetically distinct isolates, and developing an understanding of the possible virulence mechanisms of the bacterium.

## GENOME ANNOUNCEMENT

Bordetella hinzii, which has been recognized as a distinct species only since 1995 (1), is most frequently isolated from poultry. Although the bacterium was initially regarded as nonpathogenic in avian hosts, some strains induce mild to moderate respiratory disease in turkeys (2). B. hinzii is also an opportunistic human pathogen and has been associated with respiratory disease, septicemia, and cholangitis in immunocompromised individuals (3–10). It additionally infects rabbits and rodents and appears to be pathogenic in some instances (11–14). PvuII ribotyping of 22 poultry isolates, five human isolates, and one rabbit isolate provided no evidence for host-specific genotypes (14) (K. B. Register, unpublished data). Little is currently understood regarding the virulence mechanisms of B. hinzii. Of the virulence factors conserved among the classical Bordetella (B. pertussis, B. parapertussis, and B. bronchiseptica), filamentous hemagglutinin, pertactin, and dermonecrotic toxins appear to be absent in B. hinzii; most remaining Bordetella virulence factors have yet to be fully evaluated. Here, we report the genome sequences of six isolates of B. hinzii acquired from a variety of hosts and representing the four currently known PvuII ribotypes. Three isolates were obtained from turkeys, two from humans, and one from a rabbit. Two isolates were obtained in Europe and the remaining four in the United States (Table 1). These isolates are available to the scientific community through the USDA-Agricultural Research Service NRRL Collection.

**TABLE 1:** Strain description and genome assembly characteristics

B. hinzii strain	Host	No. of contigs	Total no. of bases	GenBank accession no.
1277	Human	110	4,873,106	JHES00000000
4161	Turkey	58	4,861,371	JHER00000000
5132	Rabbit	46	4,924,621	JHEQ00000000
CA90 BAL1384	Turkey	255	4,899,089	JHEO00000000
L60	Human	263	4,904,121	JHEN00000000
OH87 BAL007II	Turkey	40	4,885,897	JHEM00000000

Genomic DNA was prepared (15) and sequenced using a combination of 3- or 5-kb mate-pair Illumina MiSeq 2 × 250-bp and HiSeq 2000 1 × 100-bp paired-end reads. After quality trimming, the reads for each strain (between 1,963,892 and 7,783,417) were used for assembly with the Celera Assembler 6.1 (16) or Velvet Assembler (17). The underlying consensus sequences and gaps were improved using custom scripts to recruit unmapped reads. All the genomes have between 40 and 97 contigs, with an *N*_50_ ranging from 110,467 bp to 328,165 bp (Table 1). The overall G+C content is ~66.82%, with genome sizes ranging from 4.83 Mb to 4.92 Mb. The genomes were annotated using the J. Craig Venter Institute (JCVI) prokaryotic annotation pipeline and contain between 4,535 and 4,700 predicted protein-coding genes. The pangenome of the species was estimated at 5,882 genes, with just over 3,955 genes present in all strains and nearly 85% of the genes in any given isolate shared among all the isolates. Each strain has between 37 and 269 strain-specific genes. The majority of the unique genes encode either hypothetical proteins or proteins with functions associated with phages and other mobile elements.

The genome sequence data reported here provide a basis for the identification of putative virulence factors, for an assessment of the population structure of the bacterium, and for a more accurate establishment of its relationship to other members of the genus.

### Nucleotide sequence accession numbers.

The sequences of the six Bordetella isolates have been deposited in GenBank under the accession numbers listed in Table 1.
